# The Current State-of-the Art of LRRK2-Based Biomarker Assay Development in Parkinson’s Disease

**DOI:** 10.3389/fnins.2020.00865

**Published:** 2020-08-18

**Authors:** Hardy J. Rideout, Marie-Christine Chartier-Harlin, Matthew J. Fell, Warren D. Hirst, Sarah Huntwork-Rodriguez, Cheryl E. G. Leyns, Omar S. Mabrouk, Jean-Marc Taymans

**Affiliations:** ^1^Division of Basic Neurosciences, Biomedical Research Foundation, Academy of Athens, Athens, Greece; ^2^Univ. Lille, Inserm, CHU Lille, U1172 - Lille Neuroscience & Cognition, Lille, France; ^3^Inserm, UMR-S 1172, Team “Brain Biology and Chemistry”, Lille, France; ^4^Merck & Co., Kenilworth, NJ, United States; ^5^Biogen, Cambridge, MA, United States; ^6^Denali Therapeutics Inc., South San Francisco, CA, United States

**Keywords:** LRRK2, Rab GTPase, biomarker, Parkinson’s disease, kinase

## Abstract

Evidence is mounting that LRRK2 function, particularly its kinase activity, is elevated in multiple forms of Parkinson’s disease, both idiopathic as well as familial forms linked to mutations in the *LRRK2* gene. However, sensitive quantitative markers of LRRK2 activation in clinical samples remain at the early stages of development. There are several measures of LRRK2 activity that could potentially be used in longitudinal studies of disease progression, as inclusion/exclusion criteria for clinical trials, to predict response to therapy, or as markers of target engagement. Among these are levels of LRRK2, phosphorylation of LRRK2 itself, either by other kinases or via auto-phosphorylation, its *in vitro* kinase activity, or phosphorylation of downstream substrates. This is advantageous on many levels, in that multiple indices of elevated kinase activity clearly strengthen the rationale for targeting this kinase with novel therapeutic candidates, and provide alternate markers of activation in certain tissues or biofluids for which specific measures are not detectable. However, this can also complicate interpretation of findings from different studies using disparate measures. In this review we discuss the current state of LRRK2-focused biomarkers, the advantages and disadvantages of the current pallet of outcome measures, the gaps that need to be addressed, and the priorities that the field has defined.

## Introduction

**FIGURE 1:**
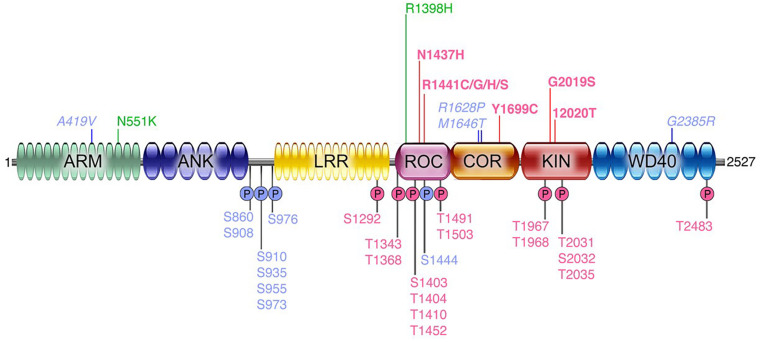
Domain architecture of human LRRK2 protein. A schematic of the known functional domains within the LRRK2 protein. Also indicated are the currently identified pathogenic mutations linked to PD (bold magenta), risk factor variants (italicized blue), protective variants (green); and below the schematic are shown several key phosphorylated residues that are auto-phosphorylation sites (pink) or phosphorylated by other kinases (blue).

Parkinson’s disease (PD) is a debilitating neurodegenerative disorder, affecting millions of people worldwide. The current therapeutic options address symptoms only and there is no approved therapy that slows progression or modifies disease course. PD is a complex disorder influenced by both genetic and environmental factors. The first unequivocal genetic data supporting susceptibility to PD were mutations found in *SNCA* (encoding α-synuclein) and the subsequent identification of *SNCA* gene duplications (Polymeropoulos et al., 1997; Singleton et al., 2003). A few years later, mutations in the leucine-rich repeat kinase 2 (*LRRK2*) gene were found to exhibit significant impact across familial and sporadic PD (Paisan-Ruiz et al., 2004; Zimprich et al., 2004). Hundreds of nonsense or missense genetic variations have been described in the *LRRK2* locus (Ross et al., 2011). However, only a few are considered pathogenic: p.Asn1437His, p.Arg1441Gly, p.Arg1441Cys, p.Arg1441His, p.Arg1441Ser, p.Tyr1699Cys, p.Gly2019Ser, and p.Ile2020Thr; with several other risk factors (e.g., p.Gly2385Arg) or variants of unclear pathogenicity (p.Arg1628Pro and p.Ser1761Arg). Their frequency varies markedly depending on the population founder effects of the G2019S-*LRRK2* mutation, reaching 30–42% of PD patients in North African Arabic populations as well as 6–30% in Ashkenazi Jewish populations, probably resulting from a mutation arising at least 5,000 years ago (for review of the genetics of LRRK2, please see Monfrini and Di Fonzo, 2017). The collective data strongly suggesting that each of the different point mutations increase kinase activity (Liu et al., 2018). Genome wide association studies (GWAS) analyses also demonstrated that variants at the LRRK2 locus, such as single nucleotide polymorphisms, are among the most important genetic risk factors for PD (Monfrini and Di Fonzo, 2017). Emerging data also suggests that intergenic LRRK2 variants may be associated with increases in LRRK2 gene expression and accelerated PD motor symptom development (Võsa et al., 2018; Iwaki et al., 2019). In Figure 1, we show a schematic of the LRRK2 domain architecture, highlighting both pathogenic as well as other risk factor or functional variants.

LRRK2 plays an important role in vesicular trafficking. It impacts endosomal, lysosomal and autophagosomal pathways (Roosen and Cookson, 2016; Alessi and Sammler, 2018), which are also affected by other well-defined PD genes, such as *SNCA* and *GBA1* (Blandini et al., 2019), strongly implicating these fundamental cellular processes in PD pathophysiology. Recent data from post-mortem PD brain and multiple *in vivo* models, suggest a role for LRRK2 in idiopathic disease as well (Di Maio et al., 2018). Preclinical studies have shown that genetic knock-out of LRRK2, inhibition of LRRK2 with small molecules, or ASO-mediated knockdown reduce pathology and protect from α-synuclein induced dopaminergic neuronal loss in rodents (Daher et al., 2014, 2015; Zhao et al., 2017), also supporting the hypothesis that, even in the absence of familial mutations, LRRK2 can be pathogenic under certain conditions.

Collectively, human genetic studies and preclinical data have led to biopharma initiating drug discovery efforts that have resulted in 2 potential therapeutics progressing into clinical trials (clinicaltrials.gov/ct2/show/study/NCT03710707; clinicaltrials.gov/ct2/show/NCT03976349). There are three potential strategies for clinical development of these LRRK2 therapeutics. Firstly, trials may selectively include genetically defined LRRK2 mutation carriers that have been diagnosed with PD. This would be dependent on patients knowing their own genetic status or focused screening efforts^1^. However, limitations in enrolling appropriate numbers of suitable LRRK2 mutation carriers will likely provide a significant hurdle in Phase 2 and Phase 3 trials; as the prevalence of *LRRK2* mutations, which are estimated at approximately 5% of all PD cases, vary significantly depending on the geographic location, and the relative frequency of specific mutations, which also varies greatly (Monfrini and Di Fonzo, 2017). If there were to be additional stratification, for example, only including G2019S or R1441C/G, this would further reduce this limited patient pool. A second potential clinical design is a prodromal approach: identifying subjects with LRRK2 mutations and determining if disease onset could be prevented by pre-treatment of the potential therapeutic. The major limitations of this approach are the limited genetic penetrance of LRRK2 (Goldwurm et al., 2011; Lee et al., 2018) and, in the cases where the mutation carriers do progress to disease, the unpredictable age of onset, as well as the absence of safety data in subjects undergoing long-term chronic LRRK2 kinase inhibition; providing significant cost/length challenges and an uncharted regulatory path.

Finally, strengthening the link between LRRK2 and idiopathic PD (iPD) could identify cohorts of patients where LRRK2, in the absence of known pathogenic mutations, is driving disease pathophysiology. In this case, the need for LRRK2 biomarkers, i.e., biological measures related to LRRK2 that can identify PD processes or therapeutic response, is absolutely critical given the heterogeneous nature of PD. As introduced above, there is evidence highlighting a link between α-synuclein and LRRK2 (Daher et al., 2014, 2015; Zhao et al., 2017). Similarly, a link has been postulated between LRRK2 and GCase activity (Alcalay et al., 2015; Nguyen and Krainc, 2018); although some controversy still exists concerning the nature of this link. Given these links as well as the prevalence of LRRK2 risk variants in the sporadic PD population, there is significant evidence supporting the therapeutic potential of LRRK2 inhibition in sporadic PD as well as additional familial PD cohorts beyond LRRK2 mutation carriers.

The clinical development of LRRK2 therapeutics will be strongly dependent on biomarkers, as target engagement and pharmacodynamic endpoints are critical for successful progression of clinical candidates (Morgan et al., 2012). This is particularly vital in PD where efficacy trials are long (average length of current trials is ∼2 years), will require significant numbers of subjects (100+ per arm), and will be costly (in the hundreds of millions USD).

There are several measures of LRRK2 function that could potentially be used in longitudinal studies of disease progression, as inclusion/exclusion criteria for clinical trials, as markers of target engagement, and as markers to predict response to therapy. Among these are levels of LRRK2, phosphorylation of LRRK2, either by other kinases or via auto-phosphorylation, *in vitro* LRRK2 kinase activity, and phosphorylation of downstream substrates or functional endpoints related to elevated (or therapeutic suppression of) LRRK2 function, which will be covered in this review.

## LRRK2 Outcome Measures

In probing the function of LRRK2, with the goal of quantifying changes that coalesce around specific disease-stratifying variables (e.g., disease state, LRRK2 mutation status, etc.), there are a number of biochemical outcome measures that are available. These include the quantification of: total LRRK2 levels; phosphorylated LRRK2 (at multiple residues; including S935, S1292, see Figure 1 and below for more details); *in vitro* kinase activity using model peptide substrates; phosphorylation of endogenous LRRK2 substrates (e.g., Rab10); and others. The specific methodologies employed for each of these measures are discussed in more detail below (see section “Assays Being Employed”). However, to date, most of the early reports (with a few exceptions, see above) assessing these targets have relied largely on Western immunoblotting, which in comparison to ELISA-based approaches for example, is limited in terms of the quantitative range that is possible, and sensitivity. Each of the measures described reveal a distinct, yet equally important, feature of the activation “state” of LRRK2; and importantly, this pattern may also manifest differently depending on the source of the biospecimen examined. Note that a summary overview of LRRK2 related measures and potential applications is given in Table 1.

**TABLE 1:** Overview of LRRK2 and LRRK2 substrate potential biomarkers and their potential use.

Potential biomarker	Current understanding	Potential use
Total LRRK2	• Expression level of LRRK2 has been shown to be modifed in disease related states, after LRRK2 kinase inhibitor treatment or after stimulation in immune cells.	• Essential to determine calculate LRRK2 phosphorylation rates. Biomarker research and exploratory studies prior to potential use in a clinical setting.
pS935-LRRK2 (rate)	• A heterologous phosphorylation site of LRRK2. Modifed in at least some disease related conditions. Signal decreases in cells exposed to LRRK2 kinase inhibitor by sensitizing LRRK2 to dephosphorylation.	• Pharmacodynamic marker in clinical trials with LRRK2 kinase inhibitors. Biomarker research and exploratory studies for assessment as PD progression or diagnostic marker.
pS1292-LRRK2 (rate)	• Autophosphorylation site. Indicator of LRRK2 kinase activity in cells. Modified in at least some disease related conditions. Signal decreases in cells treated with LRRK2 kinase inhibitors.	• Pharmacodynamic marker in clinical trials with LRRK2 kinase inhibitors. Biomarker research and exploratory studies for assessment as PD progression or diagnostic marker.
pT72-Rab8a (rate)	• LRRK2 substrate. Indicator of LRRK2 kinase activity.	• Pharmacodynamic marker in clinical trials with LRRK2 kinase inhibitors. Biomarker research and exploratory studies for assessment as PD progression or diagnostic marker.
pT73-Rab10 (rate)	• LRRK2 substrate. Indicator of LRRK2 kinase activity.	• Pharmacodynamic marker in clinical trials with LRRK2 kinase inhibitors. Biomarker research and exploratory studies for assessment as PD progression or diagnostic marker.
*In vitro* kinase assays (autophosphorylation or substrate phosphorylation)	• Indicator of intrinsic kinase activity, potentially affected by post-translational modifications.	• Biomarker research and exploratory studies for assessment as PD progression or diagnostic marker.
Genetic testing	• Pathogenic mutations and risk polymorphisms are indicators of varying degrees of increased risk for PD.	• Patient stratification.

### Total LRRK2 Levels

Total expression levels of LRRK2, depending on the tissue/cell type, can vary in PD, and thus can potentially be a useful tool to assess activation during the different stages of the disease. For example, in the CNS, LRRK2 protein levels are elevated in prefrontal cortex of PD patients (Cho et al., 2013), while CSF levels were only elevated in G2019S PD, but not in iPD or non-manifesting G2019S carriers (Mabrouk et al., 2020). Outside the CNS, immune cells are an ideal source of LRRK2 since they are obtained non-invasively and previous reports have shown elevated levels in iPD compared to healthy controls (Cook et al., 2017). In that study, levels were determined by a novel flow cytometric approach using a LRRK2 knockout validated antibody (rabbit monoclonal; clone c41-2). Specifically, LRRK2 expression was increased in CD16+ monocytes, as well as B and T cells; and this expression was correlated with both intracellular as well as secreted levels of certain cytokines (Cook et al., 2017). The regulation of LRRK2 expression in specific immune cell sub-types is unclear, however, it is known that specific pro-inflammatory mediators, such as IFN-γ, can induce expression of LRRK2 (Gardet et al., 2010); thus, the increased levels of LRRK2 in specific immune cells may be linked to elevated peripheral inflammation, which may or may not be associated with PD (e.g., Dzamko, 2017). In the earlier study of Dzamko (2017), assessing pS935-LRRK2 levels by Western immunoblotting in isolated PBMCs, no difference in total LRRK2 expression was detected between iPD and healthy control subjects in this mixed cell population. Thus, given that the bulk of LRRK2 expression in blood cells is concentrated in a few cellular sub-types (e.g., see Fan et al., 2018), including neutrophils (which were not specifically assessed in either study), it is possible that changes in LRRK2 levels, like phosphorylation of LRRK2 as discussed above, in specific types of peripheral blood cells are heterogeneous.

### Heterologous LRRK2 Phosphorylation

Phosphorylation of LRRK2 at a cluster of serine residues located within the N-terminal region of LRRK2 (e.g., S910, S935, S955, and S973), immediately upstream of the namesake leucine-rich repeat domain, represents an additional biochemical readout of LRRK2. The apparent relative abundance of these post-translational modifications (PTMs) in comparison to other sites, such as S1292, has rendered phosphorylation at these sites more easily detected, however, functional interpretation of these findings is complicated by the fact that these are not auto-phosphorylation modifications, as is the case for pS1292. Multiple kinases have been implicated in the phosphorylation of these residues, including: CK1-α1 (Chia et al., 2014), PKA (Muda et al., 2014), TBK-1 (Dzamko et al., 2012), and others. However; analogous to what is observed for auto-phosphorylation sites, phosphorylation at S935 is sensitive to pharmacological LRRK2 kinase inhibition, such that there is a rapid de-phosphorylation at this site (and the other N-terminal serine residues) following treatment of cells, or *in vivo*, with specific LRRK2 kinase inhibitors (e.g., Dzamko et al., 2010; Vancraenenbroeck et al., 2014). Interestingly, over-expressed kinase inactive mutant LRRK2 [e.g., D1994A and K1906M/R; (Ito et al., 2014)], does not display S935 dephosphorylation relative to WT, indicating that acute (pharmacological) inhibition of LRRK2 alters this regulatory cycle, while chronic genetic ablation of LRRK2 kinase activity does not. This is explained by the fact that the S935-LRRK2 phosphorylation levels do not correlate to kinase activity but rather to the sensitivity of LRRK2 to phosphatases. Indeed, LRRK2 is sensitized to dephosphorylation by LRRK2 kinase inhibitors and for certain LRRK2 mutants with reduced basal S935-LRRK2 phosphorylation. LRRK2 de-phosphorylation at the S935 cluster is mediated by the catalytic subunit of protein phosphatase 1 that is recruited to the LRRK2 complex in conditions of pharmacological inhibition of the LRRK2 kinase (Lobbestael et al., 2013). Conversely, over-expression of pathogenic mutant forms of LRRK2, such as G2019S or R1441C/G, which are known to enhance the kinase activity of LRRK2, does not show enhanced levels of pS935-LRRK2, and in fact have been reported to have decreased levels of phosphorylation at this site (Nichols et al., 2010; Li et al., 2011), including at endogenous levels in immortalized lymphoblasts from G2019S-LRRK2 mutation carriers (Dzamko et al., 2010).

The earliest report of an assay designed to quantify pS935-LRRK2 at endogenous levels came from the group of Delbroek et al. (2013). Using well-validated antibodies (i.e., in knock-out tissue), this group established a quantitative detection method for S935-LRRK2 phosphorylation that demonstrated loss of signal in kinase inhibitor-treated cells and animals. Additionally, as a proof of concept, phosphorylation at this site was detected in human PBMCs from healthy volunteers, that also was sensitive to LRRK2 kinase inhibition (Delbroek et al., 2013). Apart from the ELISA-based detection of this PTM of LRRK2, several studies employing Western immunoblotting have also been reported. The same year as the report from Delbroek et al. (2013), the group of Dzamko et al. (2013) examined pS910-LRRK2 and pS935-LRRK2 levels in PBMCs from healthy controls or iPD patients. While a significant correlation between both phosphorylated sites and total LRRK2 was found in these cells, there was no significant change in pS935 or pS910 levels, when normalized to total LRRK2, in the iPD group (Dzamko et al., 2013). The authors in this study correctly pointed out that in a mixed cellular population of PBMCs, where LRRK2 expression is concentrated in few cell sub-types (Fan et al., 2018), changes in phosphorylation of LRRK2 in distinct cellular types may not be uniform. Additional clinical studies assessing these changes in LRRK2 within specific purified cell types (e.g., monocytes, neutrophils, etc.) are necessary to determine if pS935-LRRK2 levels are detectable in selective cellular populations. In a study of a small cohort of iPD and G2019S mutation carriers, levels of pS935-LRRK2, normalized to total LRRK2 by Western immunoblotting, showed a non-significant decrease in comparison to healthy controls in isolated neutrophils (Fan et al., 2018).

### LRRK2 Autophosphorylation

The phosphorylation status of LRRK2 is reflective of its activation in a number of distinct ways. First, and most directly, *auto*-phosphorylation of LRRK2, for example at the S1292 site, is indicative of its kinase activity in the cell of origin at the time of collection. A number of factors can come into play to determine the level of phosphorylation at this, or other auto-phosphorylation site(s), not just the level of kinase activity of LRRK2 alone. For example, the presence and activity of relevant phosphatases, the sub-cellular localization of LRRK2, the activity of upstream regulators of LRRK2, the status of the ROC GTPase domain, and even the cell type, can all influence the final degree of pS1292 observed. Finally, phosphorylation at S1292 has also been detected in EVs present in CSF (Wang S. et al., 2017), at significantly higher levels, with the signal saturated in many samples, in comparison to pS1292-LRRK2 present in urinary EVs. This saturation effect in the Western immunoblot detection of pS1292-LRRK2 from CSF EVs prevented the stratification of LRRK2 G2019S carriers from non-carriers. This limitation would likely be overcome using an ELISA-based approach (with suitable antibodies for pS1292-LRRK2) in which the usable linear range of detection is typically much broader.

### Intrinsic LRRK2 Kinase Activity

Finally, in addition to the cellular indices of LRRK2 kinase activity (LRRK2 phosphorylation, phosphorylation of endogenous substrates such as Rab GTPases), the intrinsic kinase activity of isolated LRRK2 can also be informative. In this approach, LRRK2 is purified from a specific biosample (e.g., PBMCs), and an *in vitro* kinase reaction is performed using as a substrate model peptides such as LRRKtide or the related NICtide. There are several key differences between assessing kinase activity in this way (i.e., the *in vitro* activity of the purified enzyme), vs. assessing kinase activation by determining auto-phosphorylation (e.g., pS1292-LRRK2) or phosphorylation of endogenous cellular substrates (e.g., pT73-Rab10). First, performing an *in vitro* kinase reaction will allow the determination of any changes in the *intrinsic* activity of purified LRRK2. For example, it is possible that certain PTMs that are known to affect LRRK2 (e.g., phosphorylation and ubiquitination) can alter the intrinsic activity of the kinase domain. If such PTMs are more prevalent in the diseased state, compared to healthy control subjects, the functional consequence of these may be altered kinase activity. This alteration can be detected in an *in vitro* assay, in an un-encumbered way, without the potential influence of interacting proteins (depending on the stringency of the purification conditions). Secondly, “cellular” assays (measuring phosphorylation of LRRK2 or its substrates) provide a “snap-shot” of kinase activation that is the result of the coordinated action of myriad upstream and downstream regulatory factors, interacting proteins, and the general activation state of the cell. We have employed such an assay, initially in over-expression models (e.g., Leandrou et al., 2019), but more recently in a clinical study assessing LRRK2 in peripheral blood cells (Melachroinou et al., 2020). In this approach, LRRK2 is purified in an ELISA plate, capturing the protein with anti-LRRK2 (or epitope tag) antibodies, followed by an in-well kinase reaction in which the reaction mixture containing the peptide substrate is added directly to the well containing immobilized LRRK2. Possible evolutions of this *in vitro* kinase activity approach could be to include measures of autophosphorylation (such as the pS1292-LRRK2 measure, as described in Melachroinou et al., 2016) or of Rab substrate phosphorylation (by spiking in recombinant Rab substrate proteins rather than peptide substrates).

### Substrates of LRRK2 Kinase

Phosphorylation of LRRK2 substrates represents another potentially informative outcome measure of LRRK2 kinase activation; and like several of the other markers discussed, can also be dependent upon cell or tissue source. In 2016, in a landmark study from the groups of Alessi and Mann, several members of the Rab GTPase family were identified as endogenous kinase substrates of LRRK2 (Steger et al., 2016). A conserved residue within the switch II domain of these GTPases was found to be robustly phosphorylated both in cellular systems as well as *in vivo*. Several phospho-specific antibodies to certain Rab proteins have since been developed and characterized (Lis et al., 2018), and are now being deployed in studies of LRRK2 activation in clinical samples and as potential markers of target engagement. In another study, using in-house developed phospho-specific antibodies to pT73-Rab10 and pS106-Rab12, Thirstrup et al. (2017) demonstrated that a novel inhibitor of LRRK2 kinase activity could reduce phospho-Rab levels in *stimulated* PBMCs, but only after 24 h of treatment (Thirstrup et al., 2017). Likewise, similar to other reports, kinase inhibition, at 24 h, also reduced LRRK2 levels in comparison to un-treated cells. The goal in this study, as samples from PD cohorts were not examined, was to assess the utility of assessing pRab10 and pRab12 rates (i.e., phosphorylated Rab as a proportion of total Rab expression) as a marker of target engagement, and as a proof of concept study, this was indeed demonstrated. The principal caveat associated with this study is that LRRK2 levels were artificially induced in isolated PBMCs, following culture for 3 days in the presence of PMA and IFN-γ (Thirstrup et al., 2017). Later evidence demonstrated the translatability of both pS935-LRRK2 and pT73-Rab10 as pharmacodynamic readouts in the clinical setting in unstimulated PBMCs. In human subjects treated with the LRRK2 inhibitor DNL201 for 1–10 days, both readouts showed a robust exposure-dependent reduction in PBMCs (Denali Therapeutics Inc., MJFF PD Therapeutics Conference 2018).

Two studies, thus far, have examined Rab10 phosphorylation (pT73) in peripheral blood cells of PD patients, both with and without the G2019S LRRK2 mutation. A first study by Fan et al. (2018) showed the feasibility of using Rab10 phosphorylation measures by demonstrating good detection levels of Rab10 and pT73-Rab10 in neutrophils and beginning to show increases in Rab10 phosphorylation in small samples of idiopathic PD or PD with LRRK2-G2019S compared to healthy controls. In a larger study, comprised of almost 50 subjects from control or iPD groups, Rab10 phosphorylation in isolated neutrophils or PBMCs was assessed. Consistent with the earlier report, LRRK2 inhibitor treatment significantly reduced pS935 levels as well as pRab10-T73 in both neutrophils and mixed PBMC cellular populations (Atashrazm et al., 2018), with no difference in the degree of response between control and iPD subjects. Interestingly, similar to the report of increased LRRK2 expression in B or T cells, or CD16+ monocytes (Cook et al., 2017), levels of LRRK2 in purified neutrophils (but not PBMCs) are also elevated (Atashrazm et al., 2018). Additionally, neither cell type revealed differences in phosphorylation of Rab between iPD and control subjects. Taken together, while phosphorylation of Rab10, by Western immunoblotting, appears to be a suitable marker for target engagement in clinical studies of LRRK2 inhibition, it remains unclear whether this readout can reliably stratify subjects according to patient group; as thus far, differences between control and iPD or LRRK2 mutation carriers have not been observed. It should be noted, however, that for the LRRK2 mutation carrier study, the sample size and statistical power was low (intentionally, for a proof-of-concept study) precluding the possibility to reach significant conclusions. Further analyses in larger cohorts, ideally with more quantitative approaches, are clearly warranted.

## Current Assays Being Employed

An important aspect of the evaluation of LRRK2 and related targets as potential biomarkers of PD is to have a good understanding of the assays used. We present here the assay methods that have been used in recent literature (e.g., ELISA), or are at earlier developmental stages (e.g., PET tracer ligands) to measure LRRK2 status.

### Western Immunoblots to Measure LRRK2 Function

Western blots targeting pS935-LRRK2, pS1292-LRRK2, and pT73-Rab10 have been successfully used pre-clinically to detect and measure total levels of LRRK2, activation of LRRK2 kinase, and LRRK2 function. As a pharmacodynamic endpoint reflecting LRRK2 inhibition, the phospho-specific LRRK2 and Rab10 targets are well established pre-clinically. Measurement of pS935-LRRK2 showed a rapid reduction in S935 phosphorylation following LRRK2 inhibition in cellular models (Dzamko et al., 2010) and *in vivo* pharmacokinetics/dynamics studies (Delbroek et al., 2013; Fell et al., 2015; Fuji et al., 2015), enabling quantification of LRRK2 inhibitor potency in cells and tissues where LRRK2 is endogenously expressed. Similarly, both pS1292-LRRK2 (in HEK cells overexpressing a mutant form of LRRK2) and pT73-Rab10 (in mouse tissues and HEK cells overexpressing Rab10 and LRRK2) are dose-dependently reduced following LRRK2 inhibition as measured by Western blot (Sheng et al., 2012; Atashrazm et al., 2018; Fan et al., 2018; Lis et al., 2018). pS935-LRRK2 and pT73-Rab10 are also measurable by Western blot and reduced following LRRK2 inhibition *ex vivo* in PBMCs and neutrophils, demonstrating the potential translatability of these markers for clinical use (Perera et al., 2016; Atashrazm et al., 2018; Fan et al., 2018; Lis et al., 2018; and see below in section “Tissue/Biofluid Origin”).

In addition to use as pharmacodynamic readouts, several studies have measured pS1292-LRRK2 and phosphorylated Rab proteins in PD patient samples to test the hypothesis that LRRK2 kinase activity is elevated in all or a subset of PD (Dzamko et al., 2013; Fraser et al., 2016a; Atashrazm et al., 2018; Fan et al., 2018; Lis et al., 2018). pS1292-LRRK2 has not been reproducibly detectable in accessible blood matrices, while the results with pT73-Rab10 have not conclusively demonstrated elevated LRRK2 kinase activity in PD patient samples. Therefore, at this point, pT73-Rab10 (as well as pS935-LRRK2) are more likely to be useful as pharmacodynamic markers than patient selection markers, although additional studies using more sensitive and high throughput assays in additional matrices are ongoing that may change the landscape on this point.

Despite the successes of assessing LRRK2 function via Western blot in many preclinical studies; Western blot has strong disadvantages as a potential biomarker endpoint in the context of a clinical trial. In order to enable clear interpretation and quantitative analysis with rapid turnaround time, clinical assays for pharmacodynamic readouts or patient selection must be highly quantitative, ideally allowing for absolute measurement of the analyte of interest, robust to implement in different locations or over extended periods of time, and relatively high throughput. Western blots are semi-quantitative, differ greatly from user to user, and generally allow for analysis of <100 samples at a time. Therefore, new methods of LRRK2 and Rab measurement must be developed to maximize the utility of these biomarkers in the clinical setting.

### ELISA to Measure LRRK2 Function

ELISAs offer a more sensitive and high-throughput method to interrogate LRRK2 kinase activity and pharmacodynamics of LRRK2 kinase inhibitors. Thus far, three assays have been published (Delbroek et al., 2013; Henderson et al., 2015; Scott et al., 2017), each utilizing a sandwich-ELISA approach by capture with a total LRRK2 antibody, followed by detection with a specific pS935-LRRK2 antibody. The latter two studies have facilitated accurate IC_50_ measurements for LRRK2 kinase inhibitors from treated mouse tissues (brain and kidney) (Henderson et al., 2015), LRRK2 G2019S SH-SY5Y cell lysates (Scott et al., 2017), and human PBMC lysates (Padmanabhan et al., 2020). Notably, Meso Scale Discovery (MSD) has made a pS935-LRRK2 sandwich-ELISA-based assay commercially available, alongside a comparable assay that measures total LRRK2 protein levels. Use of both assays facilitates normalization of pS935-LRRK2 to total LRRK2 levels to account for compound effects on LRRK2 expression or half-life, in addition to standard normalization to tissue weight or total protein levels. These ELISAs offer enhanced sensitivity compared to Western blots, as low as 400 picomolar, as well as the option for high-throughput 384-well assay design.

An emerging alternative is the SIMOA platform offered by Quanterix, which applies digital ELISA technology. The SIMOA platform utilizes a bead-based approach to enable single molecule labeling detected by fluorescence. In addition, the SIMOA assay is able to use sample volumes often <5 μL and run up to 400 samples per shift. A recent MJFF-led study (Padmanabhan et al., 2020) utilizing the SIMOA platform, reported levels as low as 19 pg/mL for total LRRK2 and 4.2 pg/mL for pS935-LRRK2 using full-length recombinant human LRRK2; and subsequently applied this approach to human PBMC lysates. Altogether, ELISA-based assays offer a more sensitive, high-throughput alternative to Western blotting with multiplex potential for measurement of pS935-LRRK2 biomarker levels. It is crucial to note, however, that it will be vital to compare each approach, across platforms and in different centers, with parallel samples to determine if similar estimations of LRRK2 concentration and phosphorylation are obtained by the various assays.

Quantification of reduced pS935-LRRK2 by conventional ELISA and SIMOA assays can accurately reflect pharmacodynamic response following administration of LRRK2 kinase inhibitors and therefore is currently used as a surrogate biomarker, even though pS935-LRRK2 is not a direct measurement of kinase activity (see above). In fact, it has been reported that the ratio of pS935-LRRK2 to total LRRK2 is significantly reduced in human PBMC lysates from PD manifesting LRRK2 G2019S carriers compared to iPD samples and healthy controls [with and without G2019S mutations (Padmanabhan et al., 2020)], although an alternative in-house developed ELISA detected a slight but significant increase in pS935-LRRK2 in PBMCs of iPD, compared to healthy controls (Melachroinou et al., 2020). Measurement of the auto-phosphorylation site pS1292-LRRK2 would be a more ideal marker of LRRK2 kinase activity, but reliance on this biomarker has been hindered by low physiological stoichiometry (Sheng et al., 2012), and limited phospho-specific antibodies. As newer clones of antibodies targeting this site, and pT73-Rab10 as well (see above), are validated for use in more quantitative and sensitive methods such as ELISA, these challenges will likely be overcome. Nonetheless, Di Maio et al. (2018) recently reported a method using proximity ligation to amplify pS1292-LRRK2 immunostaining in the substantia nigra of human iPD tissue, which was increased compared to healthy controls. These exciting data suggest LRRK2 kinase inhibitors may have broader therapeutic potential for the larger PD patient population, beyond those carrying mutations in the *LRRK2* gene. There is potential for proximity ligation technology to be converted to more high-throughput qPCR-based platforms, though this has not yet been reported for pS1292-LRRK2. Additional improvements on the quality of reagents available for pS1292-LRRK2 detection will likely enable better utilization of this site as a biomarker. Similar quantification strategies for LRRK2-mediated phosphorylation of Rab substrates, particularly of pT73-Rab10, may also offer additional alternatives for more direct markers of LRRK2 kinase activity in the future.

### Mass Spectrometry to Measure LRRK2 Levels and Function

Liquid chromatography-mass spectrometry (LC-MS) has wide ranging applications from exploratory to regulated clinical use, yet quantitative measurements of very low abundance proteins remains challenging due to sensitivity limits and artifacts such as matrix-induced ion suppression. By and large, protein measurements by LC-MS utilize “bottom up” proteomics techniques whereby proteins are digested by proteases into smaller peptides, which are then analyzed for their signature parent and fragment ion mass-to-charge (m/z) ratios. Peptides between 10 and 20 amino acids are in the ideal range for specificity (i.e., not likely to exist in different protein types) and sensitivity (i.e., are more likely to perform better in electrospray ionization-MS). Trypsin, which cleaves proteins at the c-terminus of arginine (R) and lysine (K), is the most commonly used protease in this context. In some instances, trypsin does not yield an appropriate peptide when a specific amino acid sequence is desired. For example, a recent article by Wang S. et al. (2017) showed detection of total LRRK2 and pS1292-LRRK2 by LC-MS using the Glu-C protease since trypsin would not generate a viable peptide containing S1292 - the S1292 site is flanked by K residues (KLSK), thus trypsin would generate a 3 amino acid peptide (LSK). A peptide this short would not necessarily only come from LRRK2 and so assay specificity would be lost. The group instead chose to use the less common protease Glu-C, which cleaves at the C-term of glutamic and aspartic acid residues and this process generated a 14 amino acid peptide between E1287 and E1301 (MGKLSKIWDLPLDE) containing S1292. The mass spectrometer can then distinguish and quantify the un-phosphorylated and phosphorylated peptide species. The group then showed that phosphorylated rLRRK2 (including pS1292) was reliably detected, however, the article stops short of quantifying pS1292 LRRK2 in biological samples. It is likely that an antibody enrichment step would still be required in biological samples to be successful since no cleanup step was applied.

Although there are a number of sample cleanup steps that can reduce sample complexity (including sample fractionation), these steps can be laborious and can introduce variability. Gaining momentum in the field of protein biomarker quantification is the so-called “hybrid ligand binding assay (LBA)-LC-MS” methodology, whereby proteins are isolated from samples using antibodies (similar to ELISA), followed by protease digestion and LC-MS analysis. This methodology has the advantage of greatly reducing sample complexity and improving MS analysis. When a high-resolution mass spectrometer such as an orbitrap or FT-ICR system is used, specificity of signal is encoded by unique peptides that only exist in the targeted protein. This is an advantage over traditional ELISAs where detection specificity must be demonstrated experimentally by analyzing samples in various matrices and testing KO tissues, for example. To our knowledge there are no reported hybrid-LC-MS assays in the literature being used for routine LRRK2-pLRRK2 quantitation in the context of a fit-for-purpose biomarker assay. As this approach becomes more common, LRRK2-pLRRK2 would be well positioned for this type of assay development because of the availability of several high quality LRRK2 antibodies.

Another variation of the hybrid approach is called SISCAPA (stable isotope standard and capture by anti-peptide antibodies). This technique goes even further in reducing sample complexity. In this approach, samples containing proteins of interest are digested using a protease, and then peptides (not proteins) are isolated using anti-peptide antibodies (Anderson et al., 2004). In principle, following elution from an antibody, samples are purified for a single peptide species. In comparison, anti-protein immunocapture eluent will contain peptides from the entire protein as well as peptides from proteases used. A recent initiative by MJFF sought to develop SISCAPA based assays against regions of LRRK2 that would serve as both total LRRK2 and kinase activity endpoints. Specifically, the MJFF-SISCAPA collaboration developed mouse monoclonal antibodies against linear epitopes containing S935 (HSNSLGPIFDHEDLLK) and S1292 (MGKLSKIWDLPLD) capable of detecting both the native and phosphorylated forms of the peptides. Unfortunately, those results showed only nanogram level sensitivity, which was attributed to the performance of the target peptides on the particular LC/MS platforms used as well as the need for a higher affinity rabbit monoclonal antibody (data not published). As such, the existing assays would have limited sensitivity in the context of human CSF.

Elsewhere in this issue (Mabrouk et al., 2020), we will describe a novel SISCAPA assay using commercially available antibodies that function as anti-peptide antibodies to measure total LRRK2 with sensitivity sufficient for CSF detection.

### Development of LRRK2 PET Ligands

Positron emission tomography (PET) is a non-invasive and highly sensitive molecular imaging technique that has multiple applications across the CNS drug discovery field. For example, PET imaging with a radiolabeled molecule can be used to assess that molecule’s biodistribution properties thus allowing for the assessment of brain penetration which otherwise cannot be definitively determined in the clinical setting. PET imaging can also be used to quantify CNS target occupancy by a drug molecule and to confirm CNS target engagement. This is an incredibly powerful tool as it can determine if the hypothesis in question has been sufficiently tested in the clinic [i.e., a proof of concept (PoC) trial outcome was negative, but the CNS target was engaged sufficiently such that it rules out a role for that target in the disease/disease stage]. Finally, PET imaging has the potential to serve as a disease state biomarker if the radiolabeled molecule is specific to a target that is associated with disease or a particular stage of disease.

Given the applications of PET imaging to CNS drug discovery, the identification of a LRRK2 PET ligand could significantly enable the clinical development of LRRK2 kinase inhibitors and has been the subject of intense focus from both industry and academic groups alike. Despite the identification of numerous potent and selective LRRK2 kinase inhibitors, from a variety of structural classes, there are limited reports detailing the successful development of radiolabeled LRRK2 kinase inhibitors. In 2013, Roche/Genentech published a patent in which they described the synthesis of ^11^C- or ^18^F-labeled LRRK2 inhibitors, which were related to GNE-1023. Similarly, Wang M. et al. (2017) described the radiolabeling of [^11^C]-HG-10-102-01 but as with the Roche/Genentech probes, no *in vitro* or *in vivo* PET characterization of this molecule was described. Malik et al. (2017) reported that they had successfully radiolabeled [^3^H]-LRRK2-IN-1, however, its use as a CNS PET tracer is limited by poor off-target selectivity and limited brain penetration of the base molecule. Most recently, Chen et al., 2019 reported on the development of [^11^C]-GNE-1023 and reported excellent *in vitro* specific binding of [^11^C]-GNE-1023 to LRRK2 in rat and NHP brain sections (Chen et al., 2019). However, whole-body *ex vivo* biodistribution studies exhibited limited brain uptake of [^11^C]-GNE-1023 in mice despite not being a substrate of the brain efflux transporter Pgp. The authors reported that studies in higher species such as NHP and the development of tracers with improved brain penetration were ongoing.

Additionally, GNE-1023 has been labeled with [^18^F] rather than [^11^C], however, minimal specific binding in caudate putamen homogenates from rat, rhesus monkey, and human was reported (Zeng et al., 2018). This group also reported on studies with another radiolabeled LRRK2 kinase inhibitor (compound-B) that is derived from the indazole class and is structurally similar to the highly potent and selective LRRK2 kinase inhibitor MLi-2. While [^3^H]-compound B showed high binding affinity to LRRK2 WT full-length enzyme (*K*_*d*_ = 57 pM), only modest displaceable and saturable binding of [^3^H]-compound B was observed in rhesus monkey brain CPu homogenates (*K*_*d*_ = 0.09 nM) (Zeng et al., 2018). Importantly, using either [^3^H]-compound B or [^18^F]-GNE-1023, they determined that the *B*_*max*_ for LRRK2 in the NHP and human brain was very low (∼0.4 nM) and that the resulting tracer binding potentials (*B*_*max*_/*K*_*d*_ ratio) were far below the desired *B*_*max*_/*K*_*d*_ ratio > 10 which is typically required for the successful development of CNS PET tracers (Patel and Gibson, 2008). In summary, a validated PET ligand for monitoring changes in LRRK2 is not currently available and the probability of success for developing a LRRK2 PET tracer is low, based on observed low *B*_*max*_ (<1 nM) in the CNS regions of interest.

## Tissue/Biofluid Origin

**FIGURE 2:**
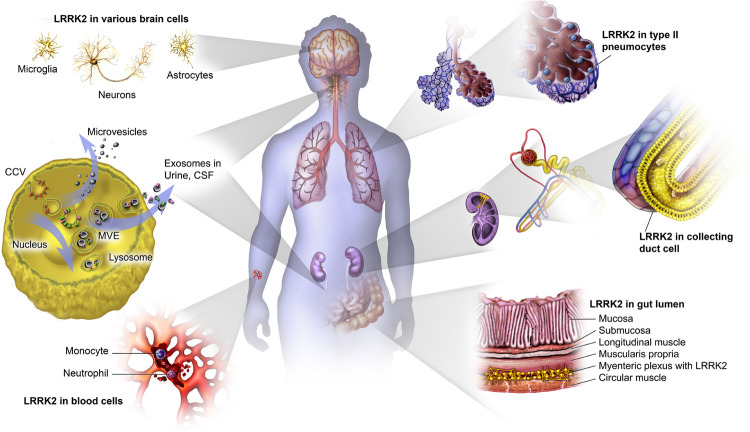
Expression of LRRK2 in multiple tissues/cell types. LRRK2 is widely expressed throughout the body in a variety of cell types and tissues, including high levels of expression in the kidney, lung, and cells of the peripheral immune system; but also in multiple brain regions, the intestine, as well as extracellularly *via* exosomal release.

LRRK2 and related measures have already been assessed as biomarker in a wide variety of tissues and biofluids (see Figure 2). Here, we provide an overview of key findings for: blood, urinary exosomes, CSF exosomes, and gut/saliva.

### Measurement of LRRK2 in Blood and Blood Derivatives

Despite LRRK2 being connected most closely with a disorder of the central nervous system, LRRK2 expression levels are highest in the periphery, in particular white blood cells (Fuji et al., 2015). This enables measurement of LRRK2 markers in blood or cells derived from blood such as PBMCs, a practical and accessible matrix in the context of clinical applications where frequent sampling for pharmacokinetics/dynamics analysis will be required. Indeed, many groups have successfully measured LRRK2 inhibition in PBMCs *ex vivo* from human samples, and in some cases *in vivo* in cynomolgus monkeys treated with LRRK2 inhibitors, by quantifying LRRK2^*pS935*^ reduction (Delbroek et al., 2013; Fuji et al., 2015; Perera et al., 2016). After the discovery that LRRK2 phosphorylates several Rab GTPases, phospho-specific antibodies targeting the LRRK2-dependent Rabs were developed and used to measure LRRK2 inhibition in PBMCs, in particular pT73-Rab10 (Steger et al., 2016; Fan et al., 2018; Lis et al., 2018). PBMCs are commonly isolated in labs and clinical sites for many applications, and are therefore clearly translatable for the purposes of measuring LRRK2 inhibition in human subjects.

LRRK2 expression varies among the different cell types of cells within PBMCs. It is most highly expressed in neutrophils and monocytes, with lower expression in T cells, B cells, dendritic cells, and natural killer cells (Fuji et al., 2015; Fan et al., 2018). This heterogeneity in LRRK2 expression combined with heterogeneity of cell populations from person to person may add to variability of LRRK2 marker quantification in PBMCs. It has therefore been proposed that isolation of neutrophils and/or monocytes for the purposes of measuring LRRK2 markers may reduce inter- and intra-subject variability, and at least in the case of neutrophils, isolation from many donors and measurement of LRRK2 inhibition either by pS935-LRRK2 or pRab10 measurement has been successfully performed (Fan et al., 2018).

In the clinical setting, it is likely that most centers will have more experience isolating PBMCs, compared to specific sub-types such as neutrophils or monocytes, so the practicalities of cell isolation must be balanced with theoretical gains of isolating a pure and homogeneous cell population. With respect to practicality, the most ideal solution for clinical measurement of LRRK2 inhibition in the periphery would be to measure it in whole blood rather than a population of cells isolated from whole blood. This would make the assay more broadly applicable and practical for clinical sites, however, the ability to track changes in LRRK2 activation within specific cell types will be sacrificed. For this reason, we developed an ELISA-based assay of pS935-LRRK2 and total LRRK2 with sufficient sensitivity for detection of these analytes in whole blood (Denali Therapeutics Inc., MJFF PD Therapeutics Conference 2018). This has indeed resulted in more practical and streamlined sample collection processes for clinical sites, compared to PBMC isolation, that are applicable for multi-center, international studies. Alternatively, at sites with such capabilities, immortalization of lymphocytes might be a useful strategy to identify new biomarkers from one type of cell. For instance, we were able to detect centrosomal cohesion deficits in PBMC derived lymphoblastoid cell lines from LRRK2 G2019S Parkinson’s disease patients, as well as in a subset of sporadic PD patients (Fernandez et al., 2019). This approach, however, is better suited for patient stratification purposes in clinical research studies, as compared to rapid and sensitive markers of target engagement required in a clinical trial.

Thus far, we have only been considering measurement of LRRK2 in blood for the purposes of target engagement, but there has been considerable effort put into measurement of LRRK2 levels and LRRK2 function in blood for the purpose of patient stratification or testing the hypothesis that PD patients without LRRK2 mutations have elevated LRRK2 function that contributes to PD pathogenesis. Total LRRK2, pS935-LRRK2 and pT73-Rab10 have all been measured in PBMCs and in neutrophils in sporadic PD patients, non-PD controls, and LRRK2 carriers with and without PD (Dzamko et al., 2013; Atashrazm et al., 2018; Fan et al., 2018; Melachroinou et al., 2020; Padmanabhan et al., 2020). LRRK2 S935 phosphorylation rates decrease in LRRK2 carriers with PD, while all other groups show no significant differences in levels of the tested analytes (Padmanabhan et al., 2020). In another study, however, pS935-LRRK2 levels were reported to be slightly elevated in iPD patients (Melachroinou et al., 2020). To be fair, for this purpose, one must consider the most relevant cell type in which to measure LRRK2 markers. In particular, in at least one report, specific monocyte sub-types have elevated LRRK2 in PD patients and release inflammatory cytokines to a greater extent in PD patients than in healthy controls following stimulation (Bliederhaeuser et al., 2016; Cook et al., 2017). Given this connection with disease, it is possible that in studies focused on patient stratification, purified monocytes may be the most relevant cell population to examine when developing blood-based markers of increased LRRK2 pathway activity in PD. Thus far, a broad characterization of LRRK2 markers or expression in monocytes in well-powered groups of PD, non-PD, and *LRRK2* mutation carriers with or without PD has not been undertaken.

### Urine-Derived Exosomes

LRRK2 is present in exosomes, i.e., cell-derived extracellular vesicles (EVs) of 30–100 nm in diameter, in several biofluids including urine ((Fraser et al., 2013) and our own results, Mutez et al., 2016). Proteomics screens of exosomes isolated from urine first indicated the presence of LRRK2 in urinary exosomes (Gonzales et al., 2009). Subsequently, the development of sensitive and specific anti-LRRK2 antibodies allowed the confirmation of the presence of phosphorylated LRRK2 in urinary exosomes.

Semi-quantitative western blot analyses of urinary exosomes have determined that LRRK2 is present in the high pg/ml to low ng/ml range (close to 1,000 pg/ml). Double immunofluorescence labeling of extracellular vesicles with anti-LRRK2 and the exosomal marker TSG101, confirmed the identity of the vesicles containing LRRK2 (Fraser et al., 2013). In light of the gain of toxic kinase function hypothesis in Parkinson’s disease, measures of LRRK2 kinase function are of particular interest, for instance the measure of LRRK2 autophosphorylation sites, including the S1292 site that has robustly been confirmed on endogenous LRRK2 in model systems as well as in human samples. Testing of LRRK2-S1292 phosphorylation in urine has revealed significantly elevated pS1292 levels in subjects harboring the G2019S mutation (Fraser et al., 2016a). This study also reported that for subjects with the G2019S mutation, S1292 phosphorylation is elevated in groups with PD symptoms compared to those without. In a separate study, the same group showed that S1292 phosphorylation is significantly increased in idiopathic PD compared with matched healthy controls (Fraser et al., 2016b). Interestingly, this study also revealed that the severity of cognitive impairment correlates with increased S1292 phosphorylation. Furthermore, a third study by the same lab examined LRRK2 in urinary exosomes compared to CSF exosomes of the same individuals and found that S1292-LRRK2 phosphorylation increases observed in urinary exosomes in subjects harboring the G2019S mutation is reflected by a similar increase in CSF (Wang S. et al., 2017). Interestingly, the study also observes that S1292-LRRK2 phosphorylation is significantly higher in CSF compared to urine in all subjects, suggesting a higher activation level of LRRK2 in brain compared to urine, and highlighting the need for more quantitative measures of LRRK2 functions.

These observations are consistent with the hypothesis that LRRK2 in urinary exosomes is modulated in disease, warranting further study of LRRK2 as a biomarker in this biofluid. For instance, the published results show a partial overlap in the distribution of S1292 phosphorylation levels in urinary exosomes in control and mutant/disease groups, suggesting that it is not an absolute predictor of disease. Also, it remains to be elucidated whether some of the observed differences are specific to certain ethnic groups or are (co-) dependent on additional factors such as dietary habits or sleep patterns, or additional lifestyle factors such as occupation. Weaknesses of this approach include the fact that it is impossible to know the cell type(s) or tissues of origin for the recovered EVs present in urine; however, given the high level of LRRK2 expression in the kidney, it is likely that much of the LRRK2 detected in these samples arises from these cells. Additionally, it is possible that more subtle changes in pS1292-LRRK2 levels may be overlooked due to the reduced sensitivity and quantitative limitations inherent to Western immunoblotting.

Besides measuring LRRK2 and its phosphorylation, other proteins in the LRRK2 complex or LRRK2 pathway offer additional possibilities as LRRK2 related biomarkers of disease or pharmacodynamic response. Therefore LRRK2’s substrates, such as the ezrin–radixin–moesin family of proteins (Jaleel et al., 2007), microtubule affinity-regulating kinase 1 (MARK1) (Krumova et al., 2015), endophilin A (Matta et al., 2012), or Rab proteins (Steger et al., 2016), are also potential biomarkers.

LRRK2 or LRRK2 pathway proteins in urinary exosomes also offer the possibility of monitoring pharmacodynamics response to potential LRRK2 targeting therapeutics. According to this hypothesis, pS1292-LRRK2, pS935-LRRK2 or phospho-Rabs would be reduced in urinary exosomes following LRRK2 inhibitor treatment. This hypothesis remains to be confirmed in biofluids. A caveat to the potential use of this biospecimen source in target engagement measures is that it was initially shown that LRRK2 release in exosomes was sensitive to pharmacological kinase inhibition, specifically *via* its interaction with 14-3-3 (Fraser et al., 2013). Thus, in samples from subjects undergoing LRRK2 kinase inhibitor treatment, the detection of exosomal LRRK2 will likely be impaired.

It should be noted that these studies also revealed sex differences in LRRK2 levels in urinary exosomes. Most notably, total LRRK2 levels are found to be higher in male compared to female subjects (Fraser et al., 2016b). In addition, pS1292-LRRK2 median levels were higher in men compared to women, while the relative elevation in pS1292-LRRK2 levels for PD versus healthy subjects is greater in women than in men. Interestingly, in a different sample set from a Norwegian patient cohort, sex differences displayed a different trend with males harboring the G2019S mutation showing higher pS1292-LRRK2 levels while the opposite holds true for females (Wang S. et al., 2017).

### CSF Exosomes

LRRK2 is not thought to exist as a soluble protein in CSF, which presents a challenge when interrogating its function in the CNS. Despite this obstacle a number of studies have demonstrated LRRK2 detection in CSF after isolating small extracellular vesicles through techniques such as differential ultracentrifugation (e.g., Fraser et al., 2013; Wang S. et al., 2017). For instance, Fraser et al. (2013) showed that in neat CSF, LRRK2 is not detectable, nor in the supernatant of ultracentrifuged CSF, but only in the pellet which contains small EVs (exosomes). Following exosome enrichment, this group has successfully applied western blotting techniques to detect total LRRK2 and pS1292 LRRK2 signals and they continue to study the biological mechanism whereby LRRK2 is introduced into these vesicles. An interesting point is that CSF pLRRK2 does not appear to correlate with urinary pLRRK2 levels and CSF levels did not correlate with disease severity while urinary levels did (Wang S. et al., 2017). It should be noted that the CSF pS1292-LRRK2 levels became saturated (within the semi-quantitative linear range of the Western immunoblot approach) compared to urinary exosomes, complicating the analyses of potential correlations with clinical features.

In terms of having a reliable biomarker endpoint that can be used in a clinical trial, exosome enrichment poses several challenges. Differential ultracentrifugation may be difficult to perform in a reproducible manner across different labs and volume requirements are quite high (∼1 ml). In addition, Western blotting analysis techniques are not considered amenable to the throughput and robustness requirements of a clinical trial. Therefore, additional techniques which can isolate LRRK2 in CSF without exosome enrichment/isolation (see Mabrouk et al., 2020) would be beneficial going forward.

## Future Directions and Approaches

### Nucleic Acid-Based Approaches

Genome wide association studies analyses revealed that LRRK2 polymorphisms are not only associated with PD, but also other disorders including Crohn’s disease and Leprosy pointing out the importance of the immune functions of LRRK2. Thus, one may expect that LRRK2 genotype stratification might help to better classify patients with higher risk to develop prominent immune phenotypes to orientate clinical trials and pharmacogenomics studies.

### Genome Wide Methylation

Assuming that environmental factors may have a larger impact on sporadic PD development compared to familial PD, it is surprising that no difference is found between sporadic PD and LRRK2 patients heterozygous for a LRRK2 mutation either in the methylation status of islands of the LRRK2 promoter in patient derived leucocytes (Fernandez-Santiago et al., 2015) or when investigating whole genome methylation of dopaminergic neurons generated from patient derived induced pluripotent stem cells (iPSCs). Several interpretations might be formulated to explain this result. The role of genetics and environmental factors is proposed to explain the reduced penetrance of LRRK2. It is thus possible that patients with or without LRRK2 mutations share a similar influence of environmental factors or that these unknown environmental factors influenced numerous low risk alleles in genes converging to LRRK2 pathways. Moreover, this same study also revealed an important PD associated hypermethylation occurring only upon differentiation into dopaminergic neurons of PD patients, but not somatic cells (Fernandez-Santiago et al., 2015). This shows that the epigenetic control of the differentiation into dopaminergic neurons plays a crucial role for the development of PD phenotypes. They also highlight the need for exploring the transcriptome expression profiles of sporadic PD and LRRK2 patients to identify biomarkers and other pathways of interest to help better understand the pathogenesis of PD.

### LRRK2 RNA Expression and Splicing

The *LRRK2* gene on chromosome 12p12 is composed of 51 exons. Usually, large genes are more likely to give rise to several transcripts due to alternative splicing events. The Ensembl database showing only one transcript encoding the full-length protein of 2527 AA is supported by strong biological evidence. Other transcripts encoding proteins of 1271 AA, 454 AA, 521 AA, 206 AA, or 78 AA, as well as 3 transcripts not encoding proteins have been proposed based on computational mapping, based on gene identifiers from Ensembl, Ensembl Genomes and model organism databases. With the development of new sequencing technologies, such as RNAseq, several groups have investigated the existence of LRRK2 RNA expression and/or splicing variants in the brain and other tissues. Of interest, association of quantitative trait locus (QTL) involving exons 32–33 have been found in the brain and is associated with the presence of a polymorphism rs3761863 (p.M2397T, involved in Crohn’s disease) together with two additional QTLs in liver and monocytes. Nevertheless, a 2019 study by Vlachakis et al. (2018) recently confirmed the existence of several spliced transcripts in brain occurring at different ratios according the studied brain regions. The development of large transcript sequencing technologies such as PacBio will enable a more robust mapping and reconstitution of each LRRK2 transcript structure. Such analyses have the potential to identify a specific transcript whose expression may be used as an early biomarker of PD and that might then be easily detectable in PBMCs.

### Transcriptome Analyses of LRRK2 Patients

Because of the nature of such studies (assessing changes at the transcriptional level rather than the protein level), transcriptomic analyses, in the context of PD biomarker development, are restricted to patient stratification in studies of disease severity and/or progression. These kinds of studies are not applicable to clinical trials of investigational compounds in which markers of target engagement are required. The transcriptome of blood or neurons heterozygous for LRRK2 variants has revealed numerous pathways, similar to idiopathic PD, that differ from controls. In PBMCs and dopaminergic neurons, we found a prevalent common dysregulation of translation, immune system signaling, and vesicular trafficking and endocytosis (Mutez et al., 2014). These results are sustained by other observations showing that LRRK2 controls several steps of these key mechanisms, such as the phosphorylation of several proteins of the translation machinery, the eukaryotic initiation factors 4EBP and ribosomal protein S15 (within drosophila models), and thus deregulating translation (Martin et al., 2014). However, the exact mechanism leading to the deregulation of translation remains poorly understood.

Transcriptome analyses have also highlighted deregulation of intracellular vesicle trafficking and function within the endocytic pathways. Their biological relevance has been confirmed (see above), since we know that LRRK2 phosphorylates at least 10 Rab GTPases regulating such processes as vesicular trafficking and endocytosis. The recent observations of Connor-Robson et al. (2019) study, using both transcriptome and proteome analyses, demonstrated that 25 of the 70 Rabs are deregulated, confirming a major role of LRRK2 in endocytosis.

RNAseq and microarray analyses of both PBMC and iPSC derived dopaminergic neurons have also demonstrated the strong deregulation of the “axon guidance pathway.” Ensemble of Gene Set Enrichment Analyses (EGSEA) of the integrated dataset revealed endocytosis and axon guidance as the two most significantly perturbed pathways, both of which were predicted to be inhibited in the presence of the G2019S mutation. The *LRRK2-G2019S* mutation has previously been demonstrated to disrupt axon guidance in iPSC-derived dopaminergic neurons (Sanchez-Danes et al., 2012; Reinhardt et al., 2013; Su and Qi, 2013; Borgs et al., 2016). Numerous reports using animal models confirmed deregulation of axonal guidance proteins [for review (Civiero et al., 2018)]. In addition, another analysis using single-cell transcriptional profiles of LRRK2 multipotent neural stem cells revealed neuronal lineages with signature similar to PD. Of note, among these genes, two regulate neurite extension upon down-regulation (NRSN1) or overexpression (SRRM4) (Ohnishi et al., 2017). The authors suggest that it could explain the discrepancies in the results obtained on neurite outgrowth assaying the LRRK2 role in neurite extension (Garcia-Miralles et al., 2015).

Interestingly, deregulation of transcripts linked to mitochondrial dysfunction is also observed by the above studies (Ohnishi et al., 2017). For instance, a significant up-regulation of nine mitochondrial genes was noted, emphasizing the critical role of mitochondria in the disease process. Additionally, the role of LRRK2 mutations in mitochondrial dysfunction is also reported in other PD patient-specific human neuroepithelial stem cells. Aberrations in mitochondrial morphology and functionality were evident in neurons bearing the LRRK2-G2019S mutation compared with isogenic controls (Walter et al., 2019).

Since deregulation of these pathways was also observed in blood cells, further study needs to be performed to establish whether some of these changes might be useful as PD biomarkers, giving clues to the development of novel neuroprotective therapeutics. In this context, Infante et al. (2016) compared the transcriptome of carriers of the LRRK2 G2019S mutation (symptomatic and asymptomatic) as well as PD patients without the G2019S mutation and controls. These comparisons highlighted six deregulated genes that were previously associated to PD risk in Genome-wide association studies (Do et al., 2011; Rhodes et al., 2011; Pankratz et al., 2012; Nalls et al., 2014). Among the 58 genes deregulated in both idiopathic PD and LRRK2 patients, those involved in oxygen transport function or iron metabolism were significantly enriched as we previously noted (Mutez et al., 2011; Mutez et al., 2014). Cell adhesion molecule perturbations were also noted in these latter studies. The deregulation of the extracellular matrix (ECM) was also noted in transcriptome profiles of iPSC derived midbrain-patterned astrocytes from PD patients harboring the LRRK2 G2019S missense mutation (Connor-Robson et al., 2019). These data put forward the involvement of Transforming growth factor beta 1 (TGFB1), an inhibitor of microglial inflammatory processes in murine models of PD (Chen et al., 2017), and matrix metallopeptidase 2 (MMP2), known to degrade α-synuclein aggregates (Oh et al., 2017).

### Lipidomics

Another potential alternative LRRK2 related biomarker is BMP [bis(monoacylglycero)phosphate], also known as lysobisphosphatidic acid (LBPA), which is an anionic phospholipid found exclusively on the intra-lumenal vesicles of late endosomes and lysosomes (Bissig and Gruenberg, 2013). BMP can also be secreted into biofluids, where it may be enriched on exosomes (Miranda et al., 2018) or apolipoprotein particles, like HDL (Grabner et al., 2019). BMP promotes electrostatic interactions between intralumenal vesicles and lysosomal lipases and their regulators (e.g., saposins) in order to facilitate glycosphingolipid degradation (Gallala and Sandhoff, 2011). BMP di22:6 levels increase dramatically in urine from patients with the lysosomal storage disorder Niemann-Pick type C, highlighting the translatability of this biomarker as an indicator of changes in lysosome function *in vivo* (Liu et al., 2014).

Several reports have now firmly demonstrated that LRRK2 activity modulates BMP levels in urine, providing key foundational evidence linking LRRK2 to lysosome function. Cynomolgus monkeys treated with LRRK2 inhibitors GNE-7915 and GNE-0877 for 7 and 29 days showed a dose dependent decrease in urine BMP after 29 days of dosing. This effect was recapitulated in LRRK2 KO mouse urine, demonstrating that the effect is on-target (Fuji et al., 2015). Similarly, the recent study by the group of Alcalay et al. (2019) showed that LRRK2 carriers had elevated urinary di-BMP levels, suggesting a link between LRRK2 and lysosomal function. While BMP reduction in urine represents on-target pharmacology of LRRK2 inhibitors, much work remains to fully understand the dynamics and biological significance of this biomarker. Studies of BMP reductions in urine following LRRK2 inhibitor treatment have focused on time points of maximal inhibition or on long-term recovery time points, so we do not have a good understanding of the timecourse or dose-dependence of BMP reduction relative to other measures of LRRK2 inhibition such as pS935 or pRab10 (Fuji et al., 2015). Additionally, the mechanism by which LRRK2 LOF leads to changes in species of BMP on a cellular level is currently unknown, confounding our understanding of the biological effects of changes in BMP in biofluids. Studies such as this give investigators new directions in understanding LRRK2 biology but also serve as potential biomarkers in clinical trials. Future lipidomic studies examining the relationship between LRRK2, GBA and lysosomal function will help define common mechanisms of genetic PD. In addition, other LRRK2 interactors have been discovered which may have value as biomarkers of LRRK2 function such as 14-3-3 (Nichols et al., 2010).

### Alternate Sample Types: Gut and Saliva

Besides improvements in detection or exploitation of additional markers in the LRRK2 pathway, additional avenues can be opened by studying alternate sample types. Besides urine, PBMCs or CSF, other types of human samples may be of interest to monitor LRRK2 or LRRK2 pathway proteins as disease or pharmacodynamic biomarkers. Interestingly, the presence of LRRK2 has been confirmed in both the enteric nervous system (ENS) as well as in the epithelial gut cells. In the enteric nervous system, Maekawa et al. (2017) report LRRK2 expression in the myenteric plexus of the small intestine. These may be of interest in relation to the gut-brain hypothesis of PD pathology whereby the GI tract is considered a trigger site of PD pathological processes (e.g., Santos et al., 2019). In relation to this hypothesis, alpha-synuclein positive structures can be found in neurons of the submucosal plexus of sporadic PD patients and these structures are similar in LRRK2-G2019S PD subjects (Rouaud et al., 2017). Further work will be required to establish whether LRRK2 expression in the ENS is limited to the myenteric plexus or whether LRRK2 is also expressed in the submucosal plexus or in enteric glial cells (Derkinderen, 2017). It also remains to be determined whether LRRK2 may contribute to α-synuclein pathology in the ENS and/or to the transmission of pathological α-synuclein species from the ENS to the CNS.

An additional link of LRRK2 with the gut is the expression of LRRK2 in epithelial gut cells, including Paneth cells (Zhang et al., 2015). This pattern of expression may be put into relation with the finding that genetic association studies have found LRRK2 to be a risk factor for inflammatory bowel disease (Crohn’s disease, CD) (e.g., Derkinderen and Neunlist, 2018; Hui et al., 2018; Ridler, 2018). Studies of LRRK2 KO mice have shown that LRRK2 in Paneth cells is involved in the lysosome sorting process to protect from enteric infection, pointing to a potential pathological mechanism for Crohn’s disease involving LRRK2 in Paneth cells (Rocha et al., 2015). It is also possible that LRRK2 gut expression may affect digestive tract symptoms that are very common in PD such as constipation. From the few studies focusing on non-motor symptoms, the frequency of such GI complications is similar between LRRK2-PD and iPD (e.g., Gaig et al., 2014). It remains to be elucidated whether levels of LRRK2, phospho-LRRK2 or the LRRK2 pathway proteins are affected in CD or PD at the level of the GI tract. A practical consideration here is the invasiveness of collecting gut samples for diagnostic purposes. The procedure performed *via* endoscopy is considered moderately invasive and is used on a routine basis to diagnose digestive disorders such as colorectal cancer, inflammatory bowel disease and peptic ulcer, therefore its application for Parkinson’s disease is feasible (Corbille et al., 2016).

Saliva is also considered a valuable biofluid for biomarker analysis and has specifically been highlighted for its potential for PD biomarkers. Indeed saliva is an attractive biofluid for diagnostics, especially in the elderly as it is much less invasive than other sample types. Principally, the primary use of saliva is as a source of DNA for genetic testing. Despite the growing interest of saliva as a biomarker fluid, little has been done to analyze LRRK2 protein or LRRK2 pathway proteins in saliva. Recently, proteomics analyses have uncovered that LRRK2 is detected in saliva as one of more than 2,000 confidently identified proteins (Pappa et al., 2018). Further research should now be performed to develop robust and quantifiable detection methods for LRRK2 in saliva and assess LRRK2 and phospho-LRRK2 levels in patient groups compared to controls.

## Conclusion

As we have outlined in the sections above, there are a great many options already available for the interrogation of LRRK2 and LRRK2-related pathways as tools in the clinical setting. We have summarized the current state of biomarker development and use in Table 1, and key outstanding issues are highlighted in Box 1. For example, as LC-MS instrumentation manufacturers continue to make gains in terms of sensitivity, ease of use, robustness, more discoveries will be made leading to novel biomarkers to advance clinical stage programs. Mass spectrometry will continue to play an important role both in LRRK2 biomarker discovery and LRRK2 clinical development. These techniques have also been used to identify novel phosphorylation sites on LRRK2 protein (Greggio et al., 2009). From an exploratory perspective, the evolution and adoption of LC-MS techniques has proven to be extremely powerful with the discovery of the Rab proteins as *bona fide* substrates of LRRK2 kinase activity (Steger et al., 2016), and in just a few short years, Rab10/pRab10 measurements have been introduced as a clinical endpoint in a LRRK2 therapeutic trial.

**Box 1. Outstanding issues.**

1. In general, studies reporting differences in specific biomarkers in PD patient groups compared to healthy control are still few in number. It remains therefore important to verify whether initial findings can be broadly replicated and extended to longitudinal studies.

2. Biomarker readouts have often been assessed individually, however, it is unclear whether a single biomarker will have sufficient predictive power. One potential path to resolve this issue is to develop a scoring system that would allow researchers to combine several biomarker readouts and thereby enhance predictive power.

3. Assays used to assess biomarker potential of LRRK2 and LRRK2 related measures have often been low-throughput assays in research laboratories (e.g., Western immunoblotting). For the most promising biomarkers, there remains a need for higher throughput robust assays that can be deployed broadly in clinical laboratories.

4. Our understanding of LRRK2 pathways has increased considerably in the last half decade. Besides kinase substrates that have begun to be considered, several other partners in these pathways remain to be assessed as potential PD biomarkers.

5. Similarly, LRRK2 phosphorylation has been intensely studied for a limited number of phosphosites (particularly S935 and S1292), however, it remains to be assessed what added value other less studied sites may have as PD biomarkers.

6. Besides potential LRRK2 related biomarkers that have emerged from proteomics and phosphoproteomic studies, other omics studies including lipidomics, transcriptomics have begun to point to potential additional potential biomarkers that require further assessment.

Finally, as it should be clear from the literature reviewed here, while the field has made great advances in the use of LRRK2-targeted biomarkers as measures of target engagement (i.e., for small molecule inhibitors of LRRK2 kinase), much work remains in optimizing the interpretation of these outcome measures for use in staging the disease, tracking progression, predicting pheno-conversion (in carriers of specific mutations), or as a tool to confirm the diagnosis of PD. For this aspect to be developed, larger multi-cohort longitudinal studies will be required, assessing multiple readouts for the presence of correlations with specific clinical features, at various disease stages.

## Author Contributions

All authors listed have made a substantial, direct and intellectual contribution to the work, and approved it for publication.

## Conflict of Interest

MF and CL are employees of Merck & Co. WH and OM are employees of Biogen. SH-R is an employee of Denali Therapeutics Inc. The remaining authors declare that the research was conducted in the absence of any commercial or financial relationships that could be construed as a potential conflict of interest. The handling editor declared a past co-authorship with one of the authors HR.

## Footnotes

1 https://www.parkinson.org/pdgeneration
